# The Rise and Fall of Interventional Pulmonology Procedures: Lessons in Innovation, Evidence, and Abandonment

**DOI:** 10.3390/diagnostics16131981

**Published:** 2026-06-25

**Authors:** Jean Reinoso, Prince Ntiamoah, Avantika Nathani, Atul C. Mehta

**Affiliations:** 1 Division of Pulmonary, Allergy, Critical Care and Sleep Medicine, Emory University, Atlanta, GA 30322, USA; jreinos@emory.edu (J.R.); avantika.nathani@emory.edu (A.N.); 2 Division of Interventional Pulmonology, Aurora BayCare Medical Center, Green Bay, WI 54311, USA; prince.ntiamoah@aah.org; 3 Integrated Hospital Care Institute, Department of Pulmonary Medicine, Cleveland Clinic Foundation, Cleveland, OH 44195, USA

**Keywords:** interventional pulmonology, bronchoscopy, innovation

## Abstract

Interventional pulmonology has produced numerous bronchoscopic and pleural procedures that were widely adopted but later declined or abandoned. This review examines selected techniques across diagnostic and therapeutic domains, focusing on their clinical Confirmed performance and reasons for attrition. Common drivers of decline include limited incremental benefit, high operator dependence, technological complexity, and competition from more effective alternatives. In several cases, early observational success was not sustained in randomized or real-world settings. Understanding why procedures fail is essential to guide the development, evaluation, and adoption of future technologies in interventional pulmonology.

## 1. Introduction

Interventional pulmonology (IP) has evolved through successive waves of technological enthusiasm, clinical experimentation, and frequently, subsequent retrenchment. Over the past four decades, numerous bronchoscopic and pleural interventions have emerged with compelling physiological rationale and early promise, only to later decline or disappear from routine practice. While some procedures have matured into durable standards of care, others have failed to withstand scrutiny from randomized trials, real-world feasibility, cost-effectiveness analyses, or shifts in disease management paradigms.

Understanding why IP procedures rise and fall is critical for the field’s future. Techniques that are abandoned are often framed as failures; however, they provide valuable insights into trial design, patient selection, industry dependence, operator learning curves, and the tension between innovation and evidence-based medicine. This review examines selected IP procedures that experienced initial adoption followed by decline, analyzing their indications, evidentiary foundations, publication trajectories, and reasons for abandonment. We argue that the lifecycle of these procedures reflects broader structural forces shaping the evolution of IP as a discipline.

## 2. Conceptual Framework: The Lifecycle of IP Procedures

Most IP procedures follow a predictable arc: physiological plausibility and unmet clinical need, early feasibility studies and single-center enthusiasm, rapid dissemination via case series and observational reports, industry involvement and device iteration, randomized or pragmatic trials, divergence into adoption, survival in very specific circumstances, or abandonment. Procedures rarely fail due to a single factor. Instead, decline usually reflects a convergence of modest efficacy, procedural complexity, complications, cost, reimbursement challenges, and evolving medical alternatives. We have categorized these procedures as diagnostic or therapeutic. Tables S1 and S2 provide a summary of these procedures, their strengths, weaknesses, and reasons for decline.

### 2.1. Diagnostic IP Procedures

#### 2.1.1. Trephine Lung Biopsy

The origins of percutaneous lung biopsy can be traced to the late 19th century, with one of the earliest descriptions attributed to Ernst von Leyden, who in 1883 successfully aspirated pathogenic material from the lung of a patient with pneumonia, thereby demonstrating the feasibility of percutaneous pulmonary sampling [1]. This approach evolved over the following decades, and by 1930, Martin and Ellis had formalized aspiration techniques for cytologic assessment [2].

Trephine lung biopsy emerged as an early minimally invasive alternative to open thoracotomy for pulmonary tissue acquisition, reflecting a broader shift toward percutaneous diagnostic strategies in respiratory medicine [3]. Initial applications were largely directed at peripheral pulmonary and pleural lesions, where percutaneous access allowed for tissue sampling with reduced procedural morbidity compared with surgical approaches. This paradigm evolved in the late 1960s, when the technique gained a broader clinical applicability for the diagnosis of diffuse parenchymal pulmonary diseases (DPLD) [4,5,6,7].

**Figure 1:**
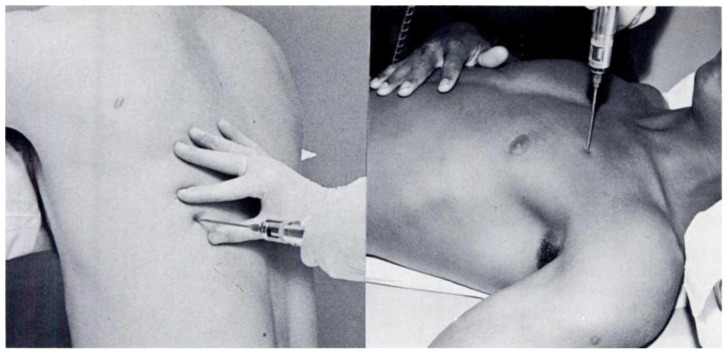
Trephine lung biopsy. A pneumatic drill-driven cutting needle used percutaneously to obtain cylindrical cores of lung parenchyma [License: 6274310453120: Copyright Clearance Center].

Trephine lung biopsy utilized a high-speed pneumatic drill coupled with a specialized cutting needle, allowing a rotating trephine to obtain large cylindrical core specimens that were substantially greater in volume than those acquired with conventional aspiration or non-cutting needle techniques, thereby improving histopathologic assessment [Figure 1]. Between 1965 and 1975, multiple institutions reported their experience with this technique. A representative series by Wilson et al. showed a diagnostic yield of 87% across 45 percutaneous biopsies in 38 patients, underscoring its clinical utility [7]. Across early published series, diagnostic yield ranged from 80% to 90% when performed by experienced operators [8,9].

Despite favorable diagnostic performance and low procedural mortality, the high complication rate limited widespread adoption. Pneumothorax was the most frequently reported complication, occurring in up to 46% of cases, with a subset requiring intervention [9]. Hemorrhagic complications were also described, and rare fatal events were reported in early series, highlighting the procedural risks associated with the technique [10,11].

Consequently, the use of trephine biopsy declined in the late 1970s. The emergence of flexible bronchoscopy and advances in computed tomography (CT) imaging facilitated a shift toward image-guided percutaneous core needle biopsy. CT-guided techniques enable precise real-time localization of pulmonary lesions and avoidance of critical structures, while maintaining comparable diagnostic yield. Contemporary series report lower complication rates, with pneumothorax occurring in 15–25% of cases and only 4–6% requiring tube thoracostomy [12].

#### 2.1.2. Closed Pleural Biopsy

**Figure 2:**
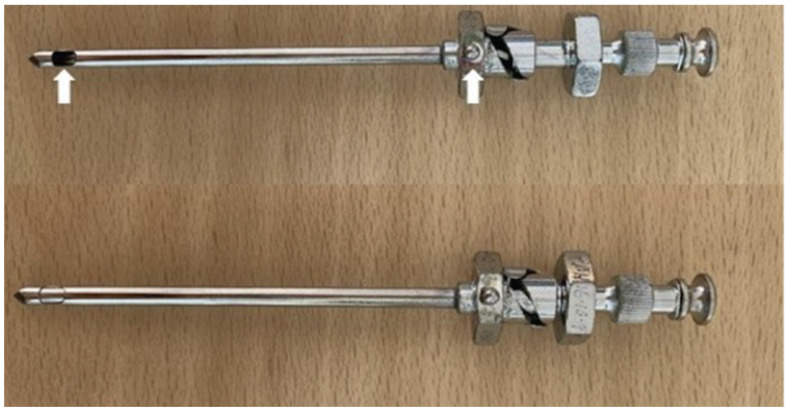
Abrams’s closed pleural biopsy needle: the top one in the open position with the notch indicator on the grip in line with the specimen open notch (arrows). Below is the needle in the closed position, as the inner cylinder has been rotated down into the outer cylinder [reproduced with permission of the © ERS 2026: Shaw JA, Louw EH, Koegelenberg CFN. A practical approach to the diagnosis and management of malignant pleural effusions in resource-constrained settings. Breathe 2023; 19: 230140 [DOI: 10.1183/20734735.0140-2023]].

Closed pleural biopsy (CPB) was introduced in the mid-20th century with the development of the Cope and Abrams needles, enabling percutaneous sampling of parietal pleura and representing a major advance in the diagnostic evaluation of pleural effusions [Figure 2]. This approach allowed clinicians to obtain tissue without the need for thoracotomy, thereby reducing procedural morbidity. Historically, CPB became the standard diagnostic modality for unexplained exudative pleural effusions, particularly in cases with negative cytology and suspected tuberculosis or malignancy, with reported diagnostic yields ranging from 35% to 80% depending on the underlying pathology [13,14].

The safety profile of CPB was favorable, with pneumothorax occurring in approximately 15% of cases, most of which did not require intervention. Additional complications included vasovagal reactions in 1–5% and hemothorax in fewer than 2% of patients [15]. However, the diagnostic performance of CPB was highly dependent on disease distribution. In malignant pleural effusions, diagnostic yield was typically below 60%, likely reflecting the focal and heterogeneous involvement of the pleura [13,16]. In contrast, substantially higher yields were observed in tuberculous pleurisy, ranging from 60% to 80%, owing to the more diffuse inflammatory involvement of the pleura [13,14,16].

The advent of medical thoracoscopy (MT) in the late 20th century marked a paradigm shift in pleural diagnostics, progressively supplanting CPB in routine clinical practice [17]. MT allows direct visualization of the pleural space and targeted biopsy of abnormal areas, resulting in consistently higher diagnostic yields, often exceeding 86% across multiple studies [18,19,20]. In addition to improved diagnostic accuracy, MT is associated with a favorable safety profile, with complication rates of approximately 10% [18]. Furthermore, when compared with video-assisted thoracic surgery, MT offers comparable diagnostic performance (93.6% vs. 96%) while reducing hospital length of stay, reinforcing its role as the preferred minimally invasive diagnostic modality in pleural disease [20,21].

#### 2.1.3. Protected Specimen Sampling

Protected bronchoalveolar lavage (PBAL) and the protected specimen brush (PSB) were developed to minimize contamination of lower respiratory tract samples introduced during bronchoscopy, particularly in the evaluation of ventilator-associated pneumonia (VAP) [22,23].

**Figure 3:**
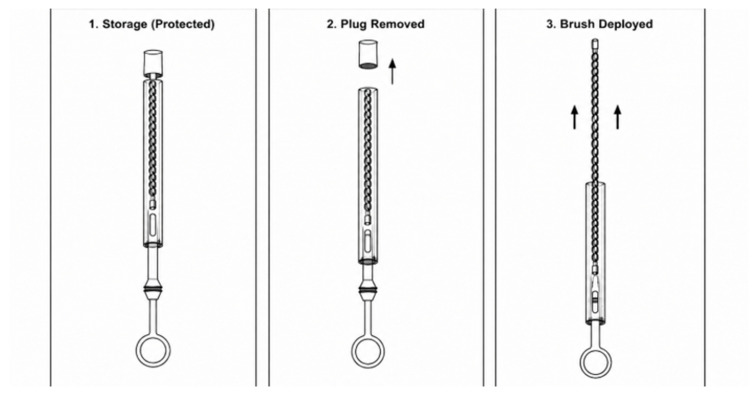
Protected specimen brush (PSB): 1. Inner cytology brush sheathed within an outer catheter and sealed by a distal polyethylene glycol plug. 2. Removal of distal polyethylene plug. 3: Brush deployed: bristles outside the catheter [original image from Dr. Atul Mehta and edited by Dr. Avantika Nathani, 2026].

PSB was first described by Wimberley and colleagues in the late 1970s as a technique designed to prevent oropharyngeal contamination during specimen acquisition [24]. The original study evaluated multiple catheter designs and demonstrated that a double-catheter system incorporating a distal polyethylene glycol plug effectively prevented contamination. In this design, the brush remained protected within an inner catheter until deployment beyond the plug, allowing sampling directly from segmental bronchi before retraction into the sheath. This approach yielded uncontaminated samples in initial testing and enabled acquisition of small-volume, highly specific specimens [25] [Figure 3].

Subsequent studies demonstrated that PSB had a sensitivity of 70–90% and a specificity of 90% for the diagnosis of pneumonia in animal models [26]. In clinical practice, diagnostic performance varied depending on prior antibiotic exposure and the quantitative culture threshold applied. In a seminal study by Torres A and colleagues, PSB achieved 100% specificity at a threshold of 10^6^ colony-forming units (CFU), though specificity declined to 59% when lower thresholds (10^3^ CFU) were used [27]. Overall, while PSB showed reasonable sensitivity in untreated pneumonia, diagnostic accuracy was significantly reduced following antibiotic administration, with false-negative rates up to 30% [22,28].

**Figure 4:**
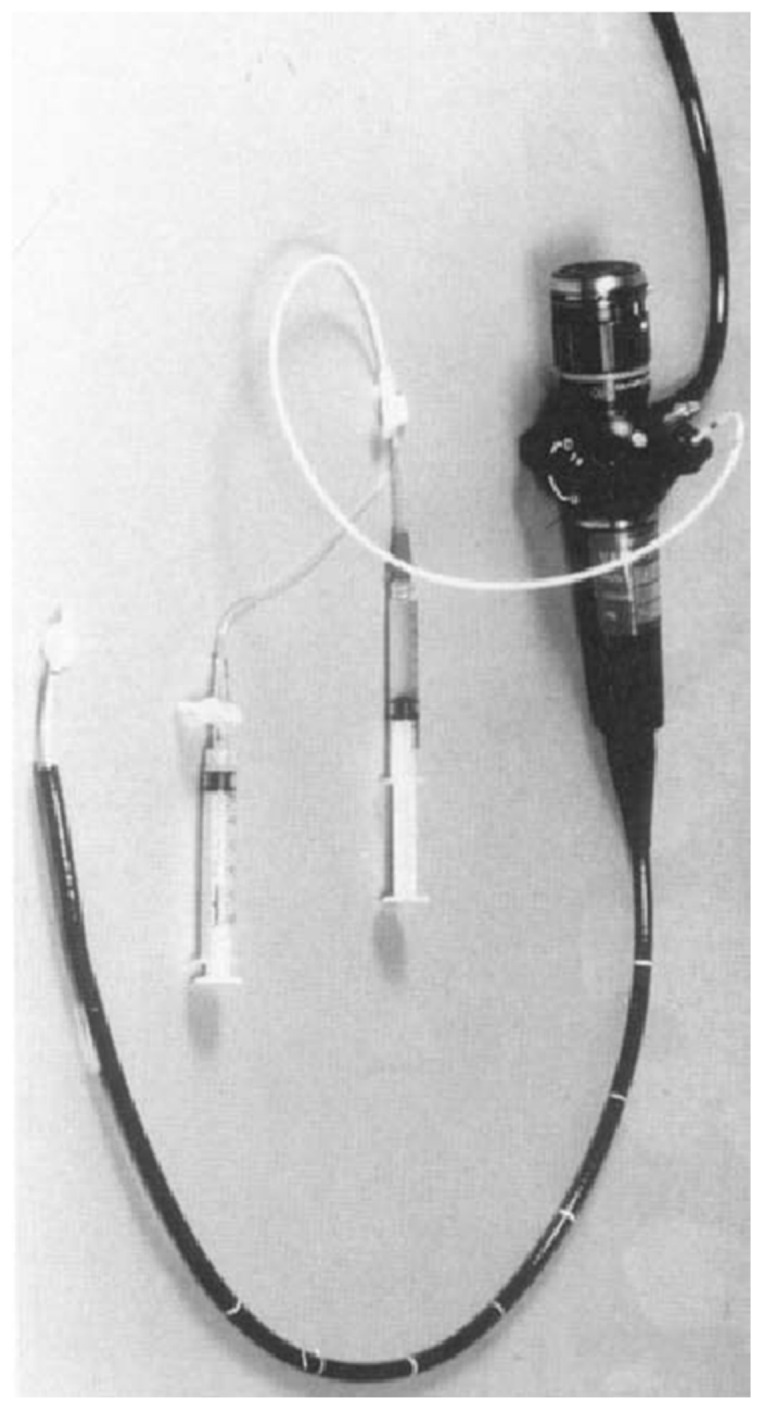
Protected bronchoalveolar lavage (PBAL): dual-lumen, balloon-tipped catheter that occludes a segmental bronchus for distal lavage and aspiration [License: 6274301309097: Copyright Clearance Center].

PBAL was subsequently introduced in 1991 as an alternative protected sampling technique. Meduri and colleagues described a balloon-tipped catheter system capable of occluding a segmental bronchus, thereby allowing lavage and aspiration of distal secretions with reduced contamination [22]. The dual-lumen design enabled simultaneous balloon inflation and fluid instillation, facilitating retrieval of larger sample volumes compared with PSB [Figure 4].

In a prospective study of patients with suspected VAP, PBAL showed a sensitivity of 87% and specificity of 91% using quantitative cultures [29]. In addition to microbiologic yield, cytological analysis, particularly the identification of intracellular organisms, provided high specificity, reaching approximately 98% when a 2% threshold was applied [29].

Despite the favorable diagnostic performance of PSB and PBAL, the use of these invasive techniques has declined. Randomized studies comparing invasive quantitative sampling strategies with non-invasive approaches have not demonstrated significant differences in clinically meaningful outcomes, including 28-day mortality, duration of mechanical ventilation, or length of intensive care unit stay [30]. Furthermore, contemporary guidelines from the Infectious Diseases Society of America recommend non-invasive sampling with semiquantitative cultures as the preferred initial diagnostic approach for VAP [31,32].

#### 2.1.4. Conventional Transbronchial Needle Aspiration

**Figure 5:**
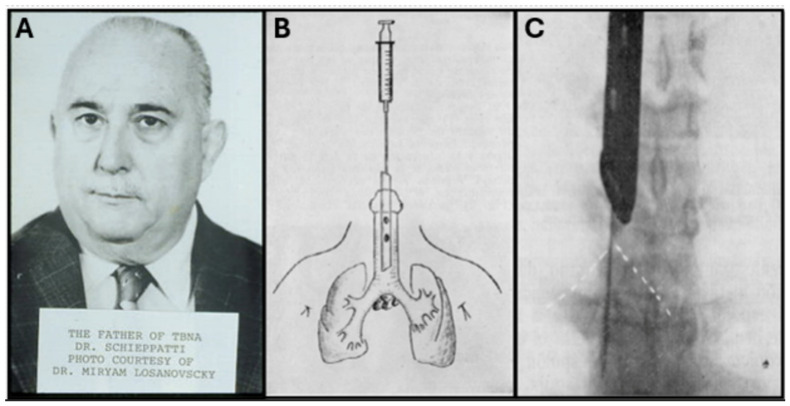
(**A**) Eduardo Schieppati, the “father of TBNA,” who first described (**B**) transbronchial needle aspiration through a rigid bronchoscope in 1949. (**C**) Conventional TBNA through a rigid bronchoscope seen on fluoroscopy [image courtesy: Dr. Miryam Losanovscky].

**Figure 6:**
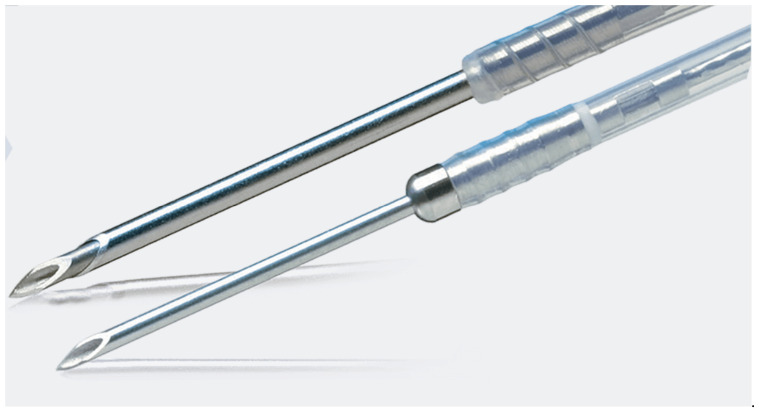
22 G Transbronchial Needle aspiration needle developed by Dr. Ko-pen Wang [image courtesy of Dr. Ko Pen Wang].

The first documented use of transbronchial needle aspiration (TBNA) is attributed to Eduardo Schieppati in 1949, who described needle aspiration of mediastinal structures through a rigid bronchoscope [Figure 5]. The subsequent introduction of flexible bronchoscopy facilitated wider adoption of the technique, and Ko-Pen Wang and colleagues demonstrated the feasibility of transbronchial needle aspiration of paratracheal lesions using flexible bronchoscopes [Figure 6] [33,34,35]. The technique was further refined with the development of systematic nodal mapping strategies, including the “Wang map,” which standardized sampling sites and established conventional TBNA (c-TBNA) as a valuable diagnostic tool for both benign and malignant mediastinal disease [35,36,37].

The diagnostic yield of c-TBNA has been reported to range widely from 53% to 90%, reflecting differences in operator experience, lymph node characteristics, and disease prevalence [38,39]. Comparative studies have demonstrated that, in selected settings and when performed by experienced operators, c-TBNA can achieve diagnostic yields approaching those of endobronchial ultrasound-guided TBNA (EBUS-TBNA) [39].

Despite these favorable results, c-TBNA remains highly operator-dependent, with optimal performance confined to centers with substantial procedural expertise. In contrast, EBUS-TBNA provides real-time visualization of target lymph nodes, enabling more accurate needle placement and access to a greater number of nodal stations compared with conventional techniques [40]. Furthermore, the design of these needles allows for deeper and more controlled sampling across the nodal capsule, improving tissue acquisition [41].

EBUS-TBNA has also been directly compared with surgical mediastinoscopy for staging of lung cancer, demonstrating comparable sensitivity and negative predictive value (approximately 81% and 91% for EBUS-TBNA versus 79% and 90% for mediastinoscopy). Accordingly, current guidelines from major societies, including the American College of Chest Physicians and the European Society of Thoracic Surgeons, recommend EBUS-TBNA (with or without endoscopic ultrasound) as the initial modality for mediastinal staging in lung cancer [42,43,44].

Nevertheless, c-TBNA may still have a role in selected clinical scenarios where comprehensive mediastinal staging is not required, such as in small cell lung cancer or granulomatous diseases like sarcoidosis, as well as in resource-limited settings where access to EBUS-TBNA is restricted [45].

#### 2.1.5. Autofluorescence Bronchoscopy

**Figure 7:**
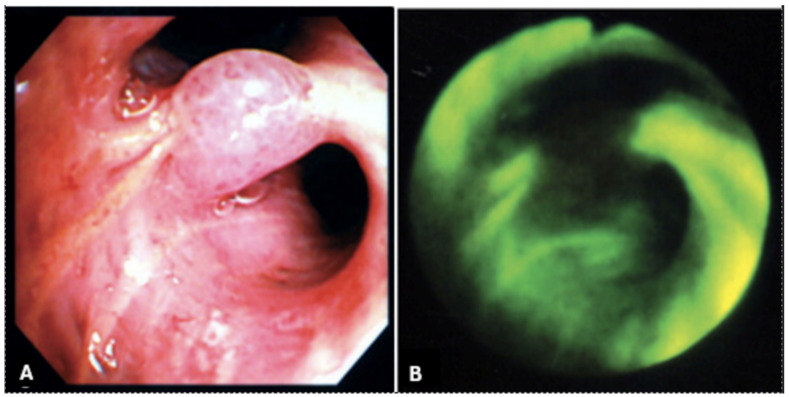
Autofluorescence bronchoscopy: (**A**) white-light view of endobronchial lesion (**B**) under blue-light illumination, normal mucosa fluoresces green while dysplastic or malignant epithelium appears red-magenta [reproduced with permission of the © ERS 2026; Chhajed PN, Shibuya K, Hoshino H et al. A comparison of video and autofluorescence bronchoscopy in patients at high risk of lung cancer. Eur Respir J 2005; 25: 951–955. [DOI: 10.1183/09031936.05.00012504]].

Fluorescence bronchoscopy was introduced in the early 1980s as a technique to enhance the detection of early neoplastic changes within the bronchial mucosa [46]. Autofluorescence bronchoscopy (AFB) is based on the emission of light from endogenous fluorophores when bronchial tissue is exposed to specific wavelengths. Under blue light illumination, normal bronchial mucosa emits a green fluorescence, whereas dysplastic or neoplastic tissue demonstrates a shift toward red or magenta hues due to alterations in tissue architecture and fluorophore composition [Figure 7] [47].

Compared with white light bronchoscopy (WLB) alone, AFB has consistently demonstrated superior sensitivity for the detection of pre-neoplastic and early malignant lesions. Two meta-analyses reported pooled sensitivities approaching 90% for AFB compared with 66% for WLB, with a significantly higher per-lesion relative sensitivity [48,49]. However, this improved sensitivity is offset by lower specificity, typically around 56% compared with 69% for WLB, largely attributable to false-positive findings arising from inflammatory changes, fibrosis, or mucous gland hyperplasia [50].

Despite its diagnostic advantages, the clinical adoption of AFB has been limited by the emergence of alternative imaging modalities. Narrow-band imaging (NBI), which can be implemented using standard bronchoscopic platforms, enhances mucosal vascular patterns without the need for specialized fluorescence equipment. In comparative studies, Herth and colleagues demonstrated that NBI offers greater specificity than AFB, with no additional diagnostic benefit when both modalities are combined [51]. More recent data from large prospective cohorts have confirmed that NBI achieves both high sensitivity (91.7%) and specificity (84.9%) in the evaluation of central airway lesions [52].

Given the need for specialized equipment, increased procedural complexity, and challenges in interpretation, the clinical use of AFB has declined. At present, its application is confined to selected cases and research settings, whereas NBI has emerged as a more practical and widely adopted modality for endobronchial lesion detection.

#### 2.1.6. Electromagnetic Navigation Bronchoscopy

**Figure 8:**
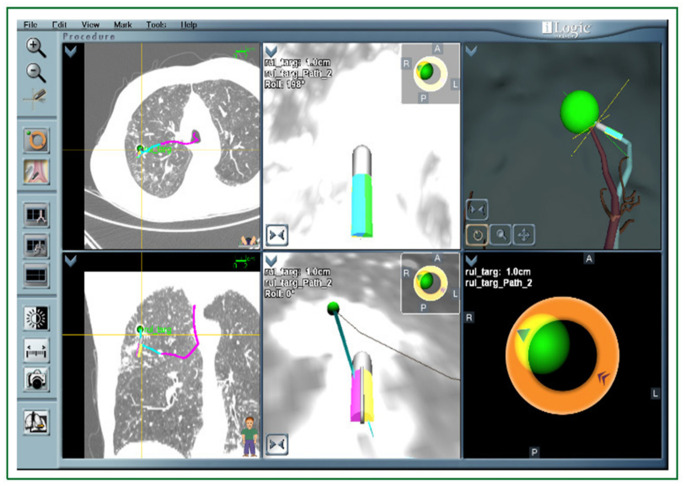
Electromagnetic navigation bronchoscopy (superDimension^®^) with extended working channel and locatable guide tracked against a pre-acquired CT roadmap [reused with permission from AME Publishing].

Electromagnetic navigation bronchoscopy (ENB) emerged in the late 1990s as an image-guided technique designed to improve access to peripheral pulmonary lesions. Early work by Stephen Solomon and colleagues demonstrated the feasibility of integrating real-time electromagnetic tracking with three-dimensional CT datasets to guide bronchoscopic navigation [53]. This concept was subsequently translated into clinical practice, and in 2006, Schwarz and colleagues reported the first use of ENB in human subjects using an early iteration of the superDimension^®^ system [54] [Figure 8].

Over the following decade, two principal ENB platforms were developed: the superDimension^®^ system (Medtronic, Minneapolis, MN, USA) and the SPiN Thoracic Navigation System (Veran Medical Technologies, St. Louis, MO, USA). The superDimension^®^ platform employs an extended working channel with a locatable guide and an external electromagnetic field generator to track the position of bronchoscopic instruments relative to a pre-acquired CT map. It remains the most extensively studied ENB system, with reported diagnostic yields ranging from 38.5% to 96.8% across heterogeneous studies [55].

The largest prospective evaluation of this platform is the NAVIGATE study, which demonstrated a diagnostic yield of 73% at 12 months and 67.8% at 24 months, with a favorable safety profile, including a pneumothorax rate of 2.9% requiring intervention and a 1.5% rate of moderate hemorrhage [56,57].

The SPiN Thoracic Navigation System incorporated a distinctive design, featuring “always-on” tip-tracked instruments with embedded electromagnetic sensors, allowing real-time tracking of both the biopsy tool and target lesion. This system also enabled electromagnetic navigation transthoracic needle aspiration, expanding access to lesions not reachable via the bronchial tree. Reported diagnostic yields for this platform ranged from 33% to 90.2%; however, more recent multicenter data have demonstrated more modest performance, with diagnostic yield around 53% when strict outcome definitions are applied [58].

More recently, technological advances have led to the development of robotic-assisted bronchoscopy (RAB) platforms, which aim to improve stability, reach, and precision in peripheral lung navigation. Comparative studies suggest that RAB is non-inferior to newer iterations of ENB systems, such as Illumisite, while offering potential advantages in procedural ergonomics [59]. Indeed, objective workload assessments have demonstrated that ENB is associated with higher cognitive demand compared with robotic systems, as measured by validated surgical task load indices [60].

With increasing adoption of robotic platforms, the use of conventional ENB systems has declined. However, the higher cost and infrastructure requirements of robotic bronchoscopy may limit widespread implementation, and ENB continues to have a role in resource-limited settings and lower-volume centers [61].

#### 2.1.7. Confocal Laser Endomicroscopy

**Figure 9:**
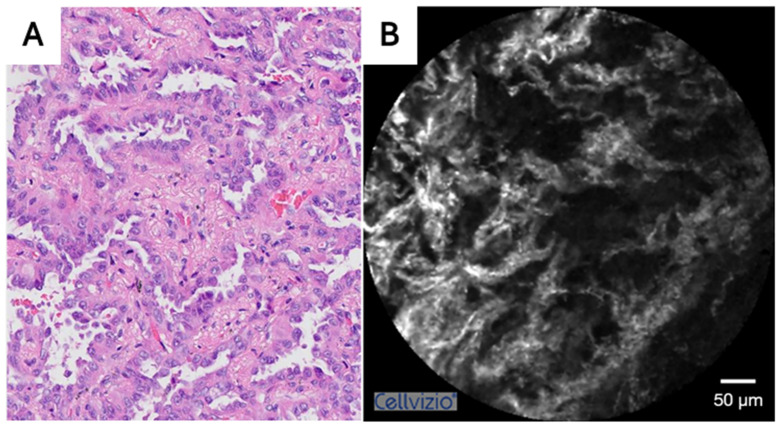
Confocal laser endomicroscopy (CLE): (**A**) hematoxylin and eosin stain of invasive lung adenocarcinoma; (**B**) CLE probe providing real-time near-histologic “optical biopsy” images with thickened alveolar structural destruction [reused with permission from AME Publishing].

Fibered confocal laser endomicroscopy (CLE) is an optical imaging technique that enables real-time, in vivo visualization of tissue microstructure at near-histologic resolution. By exploiting autofluorescence from endogenous fluorophores, CLE generates high-resolution images of cellular and extracellular architecture, thereby providing a form of “optical biopsy.” The feasibility of applying CLE to the respiratory tract was first demonstrated by Thiberville and colleagues in the late 2000s, who successfully introduced a flexible probe through the working channel of a bronchoscope to visualize airway microstructure [62] [Figure 9].

CLE has since been explored across multiple domains of pulmonary medicine, with the most relevant applications in lung cancer and DPLD. In the context of peripheral pulmonary lesions, CLE has been integrated with advanced navigation platforms, including robotic-assisted bronchoscopy, where preliminary studies suggest that real-time endomicroscopic imaging may improve targeting and sample adequacy [63]. In the realm of lung transplant, probe-based CLE was an attractive candidate to assess for acute cellular rejection in vivo. Keller and colleagues found that the use of CLE to identify increased peripheral vascular cellularity correlated with patients that had acute cellular rejection on transbronchial biopsy. Importantly they also report a non-trivial learning curve to be able to interpret CLE images [64].

Needle-based CLE has also been evaluated for real-time tissue characterization, with studies demonstrating high diagnostic accuracy for malignancy, when images are interpreted by blinded observers [65]. At the time of this publication, there is a multicenter randomized trial currently recruiting patients to needle-based CLE aided bronchoscopy vs. conventional bronchoscopy without CLE [66]. However, despite these promising applications, CLE has not been incorporated into routine clinical practice yet. In lung cancer, its current role is adjunctive, serving to guide or confirm a good position for biopsy rather than replace histopathologic diagnosis. Furthermore, alternative imaging modalities such as radial probe endobronchial ultrasound and cone beam CT, which are more widely available and easier to integrate into standard workflows, continue to serve as the primary tools for lesion localization. In DPLD, CLE may provide complementary information; however, its diagnostic value remains insufficiently validated to support routine use in clinical decision-making or multidisciplinary discussion. And lastly in lung transplant patients, although CLE is able to correlate vascular cellularity with acute cellular rejection, it has not replace transbronchial biopsy. The outcome of the upcoming needle based CLE trial [66] would be important to determine if CLE remains as an academic curiosity or it enters the IP practice as another adjunctive image modality.

### 2.2. Therapeutic IP Procedures

#### 2.2.1. Transtracheal Oxygen Therapy (TTO2T)

TTO2T was introduced by Heimlich in 1982 as a percutaneous method of delivering oxygen directly into the trachea [67]. In a follow-up report published in 1985, Heimlich and Carr described clinical experience in more than 100 patients and emphasized improved efficiency, comfort, and compliance compared with nasal cannula oxygen [68]. Subsequent studies by Hoffman and colleagues, and later by Kampelmacher and colleagues, showed that TTO2T reduced oxygen flow requirements, could improve exercise tolerance, and was generally acceptable to selected patients on long-term oxygen therapy [69,70,71] [Figure 9].

**Figure 10:**
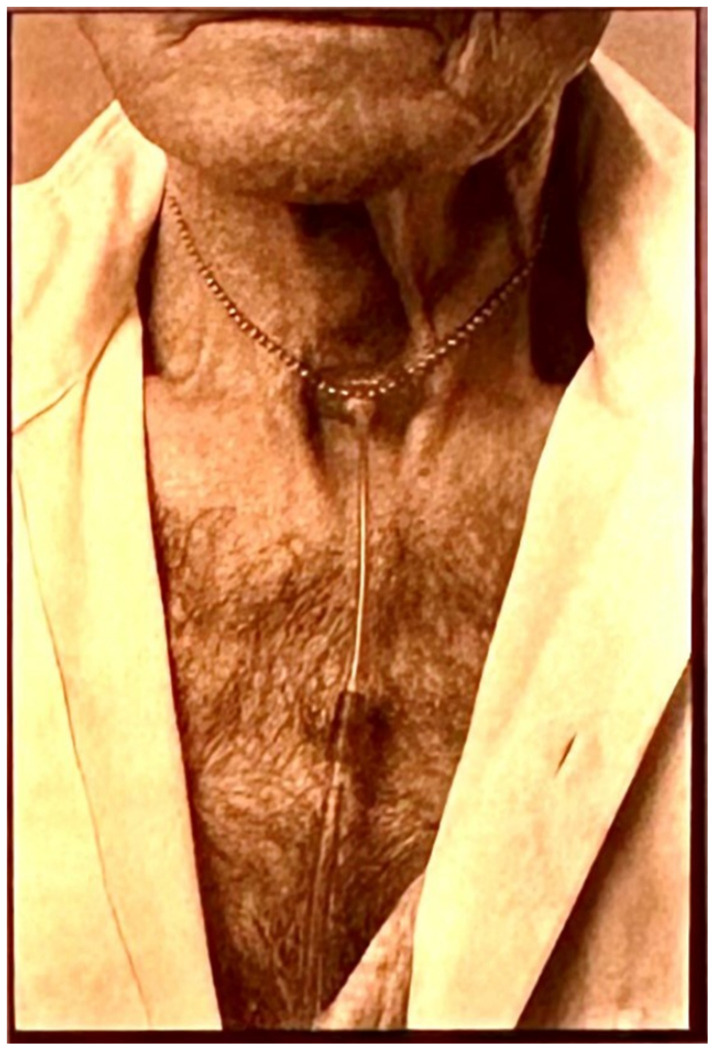
Transtracheal oxygen therapy (TTO2T): small-bore percutaneous catheter delivering oxygen directly into the mid-trachea [image courtesy of Dr. Atul Mehta].

TTO2T was developed to improve oxygen delivery efficiency by passing upper-airway dead space, thereby reducing the oxygen flow needed to maintain adequate oxygenation [Figure 10]. Early clinical studies also suggested improvements in mobility, comfort, and quality of life in selected patients [68,69,70,72,73]. Despite these advantages, long-term use has been limited by catheter-related complications and maintenance burden [74,75].

The complication profile depends partly on the insertion technique. The modified Seldinger approach became the standard technique, but Lipkin and colleagues later described a surgical “minitrach” method that allowed earlier initiation of TTO2T and more rapid tract maturation [74]. Christopher’s later review likewise noted that the modified Seldinger approach was the traditional standard, while surgically created tracts were developed to reduce tract-related problems [75].

Regardless of insertion technique, mucus plugging is the most characteristic long-term complication. In the Mayo Clinic series reported by Orvidas and colleagues, mucous plugging occurred in 38% of patients [76]. Additional case reports described partial airway obstruction from mucus-ball formation, and fatal tracheal obstruction has also been reported [77,78,79]. These events contribute substantially to the maintenance burden of TTO2T [74,75,76].

Granulation tissue at the catheter site is another important complication and may cause airway narrowing or obstruction. Punzal and colleagues reported a case requiring bronchoscopic laser resection of granulation tissue secondary to a transtracheal oxygen catheter [80]. Tracheal mucosal injury has also been reported, including ulcerative tracheitis and airway trauma associated with catheter position and mucus-ball formation [79,81].

For these reasons, TTO2T has generally remained an intervention for carefully selected, highly motivated patients who can manage daily catheter care [75,76]. Although physiologically sound, its broader adoption appears to have been limited by complication burden, tract care requirements, and the availability of simpler noninvasive oxygen-delivery systems [75,76].

The decline in TTO2T use should not be interpreted as a failure of the intervention itself. Rather, it reflects the cumulative impact of a high complication and maintenance burden, declining patient acceptance, the absence of demonstrated mortality or hospitalization benefit, and substantial advances in portable and non-invasive oxygen delivery systems. TTO2T therefore exemplifies how physiologically sound IP therapies may lose clinical relevance when complexity and long-term maintenance outweigh incremental benefits.

#### 2.2.2. Bronchial Thermoplasty

Bronchial thermoplasty (BT) emerged in the early 2000s as a novel interventional strategy for patients with severe, refractory asthma, targeting airway smooth muscle hypertrophy as a structural driver of airflow limitation and bronchial hyperresponsiveness. Unlike pharmacologic therapies directed primarily at airway inflammation, BT sought to modify airway architecture through the delivery of controlled radiofrequency energy to the bronchial wall, resulting in sustained reduction in smooth muscle mass and remodeling of airway architecture [82]. This mechanistic focus represented a conceptual shift in asthma management, particularly for patients with persistent symptoms despite maximal medical therapy [83].

Following early feasibility studies in the early 2000s, BT underwent formal evaluation in randomized controlled trials over the subsequent decade. The AIR and the RISA trials published in 2007, demonstrated reductions in asthma exacerbations and improvements in symptom control in selected patients with moderate-to-severe disease [82,84]. The pivotal AIR2 trial, by Castro et al. published in 2010 and, employed a sham-controlled design and demonstrated significant improvements in asthma-related quality-of-life scores, along with reductions in severe exacerbations and emergency healthcare utilization, albeit in the context of a substantial placebo response [85].

Long-term follow-up studies published between 2013 and 2021 reported sustained reductions in exacerbation frequency extending to 5 and 10 years after treatment, without evidence of accelerated lung function decline, progressive airway injury, or increased hospitalizations [86,87,88,89]. These findings established BT as one of the few IP procedures supported by durable randomized and longitudinal outcome data.

Despite its evidentiary foundation, BT has consistently been associated with a measurable short-term morbidity. Across trials conducted between 2007 and 2010, treatment required three bronchoscopic procedures performed over several weeks, with predictable transient worsening of asthma symptoms, bronchospasm, atelectasis, and short-term increases in healthcare utilization during the peri-procedural period [84,85,87]. Although these events were generally self-limited and resolved within weeks, they represented a non-trivial burden for both patients and healthcare systems.

Following regulatory approval in 2010, BT experienced initial uptake; however, by the mid-to-late 2010s, utilization began to plateau and subsequently decline in many regions. This trend coincided with the rapid expansion of biologic therapies for severe asthma, with biologics targeting IgE, IL-5, IL-4/IL-13, and later thymic stromal lymphopoietin pathways, many of which demonstrated superior reductions in exacerbation rates with minimal procedural risk and greater ease of administration [90].

In parallel, the eligible patient population for BT remained narrow, requiring severe disease refractory to optimized inhaled therapy and careful phenotypic selection. Variability in reimbursement, institutional resource requirements, and the need for specialized procedural expertise further limited widespread adoption. As asthma management paradigms increasingly shifted toward precision medicine throughout the late 2010s and early 2020s, BT assumed a progressively narrower role within treatment algorithms. Reserved for patients who had failed biologics [90].

BT represents a distinctive example of an IP procedure that succeeded scientifically yet lost clinical momentum over time. From its validation in randomized trials between 2007 and 2010 to and demonstration of long-term durability, BT met conventional standards for efficacy and safety [85,86,87,89]. Its subsequent decline reflects displacement rather than failure, driven by evolving therapeutic landscapes and patient preference for less invasive options.

BT illustrates a central lesson in IP: even rigorously validated procedures may lose relevance when procedural burden and scalability are outweighed by more adaptable medical alternatives.

#### 2.2.3. Endobronchial Laser Photoresection (Nd: YAG Laser)

Endobronchial laser photoresection, most commonly using the neodymium-doped yttrium aluminum garnet (Nd: YAG) laser, emerged in the late 1970s and early 1980s as one of the first effective bronchoscopic therapies for malignant central airway obstruction [91,92]. At a time when bronchoscopy was largely diagnostic, the ability to achieve rapid endoluminal tumor debulking through photothermal coagulation and vaporization represented a major conceptual advance. Nd: YAG laser therapy enabled immediate restoration of airway patency, palliation of dyspnea, and control of endobronchial bleeding, particularly in patients with unresectable lung cancer or poor surgical candidacy [91,92].

**Figure 11:**
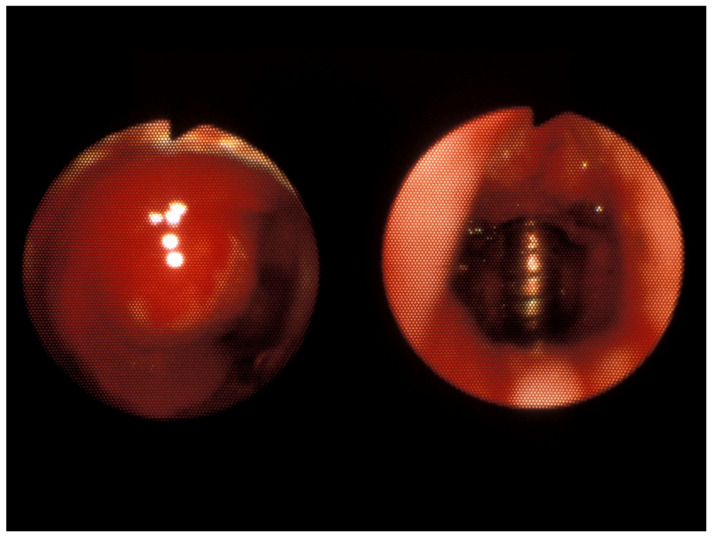
Nd: YAG laser photoresection: bronchoscopic delivery of near-infrared laser energy for coagulation and vaporization of exophytic endobronchial tumor. Note: patient was intubated with a metallic endotracheal tube, through a preexisting tracheostomy stoma [image courtesy of Dr. Atul Mehta].

Early adoption was driven by its capacity for deep tissue penetration and effective coagulation, making it particularly suited for bulky, exophytic endobronchial tumors [91,92]. The technique rapidly became central to therapeutic bronchoscopy in specialized centers [Figure 11].

Throughout the 1980s and early 1990s, observational studies demonstrated high rates of immediate airway recanalization, symptomatic improvement, and hemostasis following Nd: YAG laser treatment [91,92]. Pioneering work by Jean-François Dumon and contemporaries helped standardize technique, indications, and safety parameters, establishing laser photoresection as a foundational IP procedure [92].

However, the evidence base reflected the era in which it was developed. Most studies emphasized technical success and short-term palliation rather than survival benefit or comparative effectiveness [91]. Randomized controlled trials were scarce, and laser therapy was rarely evaluated against emerging alternatives.

Despite its effectiveness, Nd: YAG laser photoresection was associated with a non-trivial risk profile. Complications included airway fire, hemorrhage, perforation, and delayed airway necrosis [93,94]. The requirement for reduced inspired oxygen concentrations during laser activation limited its use in severely hypoxemic patients, while deep tissue penetration increased the risk of collateral airway injury [93,94].

These safety considerations confined widespread adoption to high-volume centers with specialized expertise and infrastructure, reinforcing a steep learning curve and limiting scalability [93,94].

Beginning in the late 1990s and early 2000s, the role of Nd: YAG laser diminished as other bronchoscopic modalities became more widely available. Argon plasma coagulation, electrocautery, cryotherapy, and mechanical debulking offered many of the same therapeutic goals with lower cost, simpler workflow, or a more favorable safety profile in routine practice [93,94].

In parallel, advances in cross-sectional imaging, particularly CT, enabled earlier detection and more precise characterization of central airway lesions, improving procedural planning and reducing the frequency of presentation with advanced endoluminal obstruction requiring urgent ablative intervention [94].

The decline of Nd: YAG laser photoresection does not reflect a lack of efficacy, but rather technological supersession. Laser therapy worked within its intended context. However, its procedural complexity, safety constraints, and resource intensity rendered it less compatible with the evolving priorities of IP.

In retrospect, Nd: YAG laser therapy represents a formative but transitional technology: one that defined the early therapeutic ambitions of the field yet ultimately yielded to more adaptable and safer alternatives. Its trajectory underscores a recurring theme in IP, the displacement of powerful but complex tools by technologies that are easier to apply, more cost-effective and more sustainable than those focused on maximal technical capability.

#### 2.2.4. Airway Bypass Procedures for Emphysema

**Figure 12:**
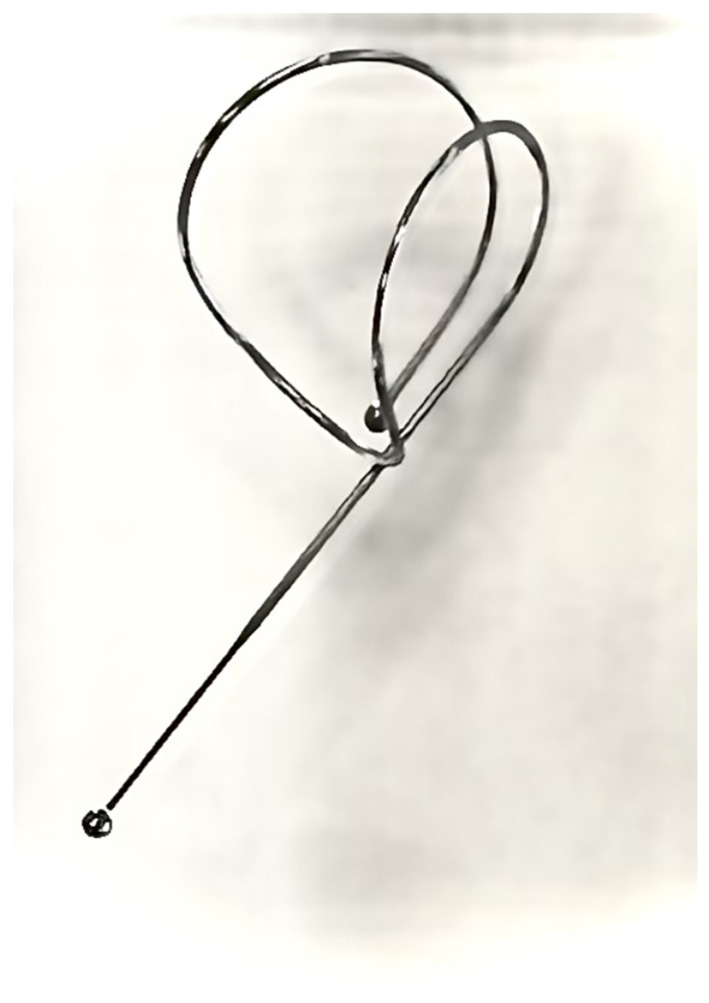
Endobronchial coil: preformed nitinol coil deployed via bronchoscope, resuming its shape to mechanically compress diseased subsegmental lung [image courtesy of Dr. Atul Mehta].

Airway bypass was developed in the early 2000s for patients with severe emphysema and hyperinflation on the premise that creating extra-anatomic passages from emphysematous lungs to central airways could exploit collateral ventilation and permit trapped gas to escape, thereby reducing hyperinflation without resection [95,96] [Figure 12].

Early feasibility work supported the concept and led to the randomized, sham-controlled EASE trial in 2011 [96]. In EASE, the procedure was technically feasible and produced early physiologic changes, but the benefits were not durable. The loss of effect was attributed to closure of the newly created tracts, including mucus plugging, inflammation, and granulation tissue, which undermined long-term patency and reproducibility [96].

Although airway bypass procedures were generally feasible, they were associated with procedural complexity and adverse events, including pneumothorax, hemoptysis, and device occlusion. The need for multiple stents and, critically, the inability to maintain long-term tract patency limited reproducibility and sustained clinical benefit [96,97].

As bronchoscopic lung volume reduction evolved, phenotype-driven approaches, particularly endobronchial valves, showed more consistent and durable benefits in appropriately selected patients [98,99]. In this context, airway bypass fell out of clinical use.

Airway bypass procedures illustrate the limitations of physiologically elegant concepts when biological response and tissue healing cannot be adequately controlled. Despite innovative design and early promise, the inability to sustain airway patency precluded clinical adoption. This experience highlights the importance of durability, not just immediate physiological effect, in determining the long-term viability of IP procedures.

#### 2.2.5. Bronchoscopic Lung Denervation

Bronchoscopic lung denervation, more precisely targeted lung denervation (TLD), was conceived as a procedural analog to anticholinergic therapy in chronic obstructive pulmonary disease (COPD). By ablating parasympathetic fibers around the main bronchi, it aimed to reduce vagally mediated bronchoconstriction and mucus secretion independent of inhaled pharmacotherapy [100,101]. This approach was supported by established physiological principles and pharmacologic success of anticholinergic agents, positioning TLD as a potential disease-modifying intervention.

Early studies showed technical feasibility, but later trials produced a more mixed picture. AIRFLOW-1 demonstrated acceptable feasibility and longer-term safety after procedural modifications, while AIRFLOW-2 suggested fewer severe COPD exacerbations over 2 years in treated patients, but the signal was modest and the broader clinical role remained uncertain [100,101].

Early-generation devices were associated with safety concerns, including gastrointestinal adverse events related to unintended injury to peribronchial vagal branches [100,101,102]. Longer-term data did not demonstrate a consistent or clinically meaningful advantage over optimized medical therapy, limiting broader adoption [102].

The problem with lung denervation was not a lack of mechanistic rationale. Rather, it entered a therapeutic landscape already being reshaped by optimized inhaled triple therapy and increasingly sophisticated COPD phenotyping. In that setting, an invasive bronchoscopic intervention with procedural complexity and only incremental benefit struggled to establish a durable role. That trajectory makes it a useful example of how even conceptually elegant interventions may falter when their effect size is not clearly superior to contemporary medical therapy. Its trajectory reinforces the need for clear superiority, or at least durable non-inferiority, when introducing invasive interventions into chronic disease management.

#### 2.2.6. Early Endoscopic Lung Volume Reduction Precursors

Prior to the success of valve-based lung volume reduction, several bronchoscopic approaches were developed to replicate surgical lung volume reduction. These included biological sealants (glue), thermal vapor ablation, and endobronchial coils, each designed to reduce hyperinflation through parenchymal collapse, remodeling, or mechanical compression [103,104].

#### 2.2.7. Sealants and Biological Glue

Polymeric lung sealants such as AeriSeal were intended to occlude distal airspaces and induce remodeling and volume loss. Early studies showed that this approach could reduce hyperinflation and improve symptoms in selected patients, but inflammatory reactions, COPD exacerbations, and concern about irreversibility limited broader adoption [103,105].

#### 2.2.8. Vapor Ablation

Bronchoscopic thermal vapor ablation deliberately induces a localized inflammatory reaction to achieve segmental volume reduction. In the STEP-UP trial and related analyses, vapor therapy improved lung function, and quality of life in carefully selected patients with upper-lobe predominant emphysema, including some with collateral ventilation, but treatment-related inflammation and strict selection requirements remained major constraints [106,107].

#### 2.2.9. Coils

Endobronchial coils reduce hyperinflation by mechanically compressing diseased lungs and do not depend on absent collateral ventilation [Figure 12]. Randomized studies such as REVOLENS and RENEW demonstrated modest improvements in exercise capacity, lung function, and quality of life, but these gains were accompanied by higher adverse event rates and a less favorable risk–benefit profile than initially anticipated [104,108,109]. Designed to be a reversible procedure was later recognized to be otherwise further limiting its applicability.

Taken together, these precursor approaches were important not because they became durable standards, but because they clarified the principles that later defined successful bronchoscopic lung volume reduction: careful phenotyping, predictability of response, and an acceptable trade-off between efficacy and procedural burden. Their decline therefore reflects iterative refinement rather than failure [99,110].

## 3. Conclusions

Collectively, these trajectories illustrate that IP advances not through linear progress, but through iterative experimentation, selective abandonment, and integration of lessons learned. Overall, these 12 procedures we have reviewed have failed or declined due to different factors: complication burden such as the case of trephine lung biopsy; modest or non-sustained effects in a pragmatic setting in the case of PSB and PBAL, displacement for a more scalable alternative or a shift in the therapeutic landscape in the case of bronchial thermoplasty.

Procedures that decline are not failures, but filters, refining patient selection, trial design, and mechanistic understanding. Recognizing these patterns may help the field align innovation more closely with durable clinical impact.

## Data Availability

No new data were created or analyzed in this study. Data sharing is not applicable to this article.

## Supplementary Materials

The following supporting information can be downloaded at: https://www.mdpi.com/article/10.3390/diagnostics16131981/s1, Table S1: Rise and Fall of Diagnostic Interventional Pulmonology Procedures; Table S2: Rise and Fall of Therapeutic Interventional Pulmonology Procedures.

## Abbreviations

AFB	Autofluorescence bronchoscopy
BT	Bronchial thermoplasty
CFU	Colony-forming units
CLE	Confocal laser endomicroscopy
COPD	Chronic obstructive pulmonary disease
CPB	Closed pleural biopsy
CT	Computed tomography
DPLD	Diffuse parenchymal lung diseases
EASE	Exhale Airway Stents for Emphysema (trial)
EBUS-TBNA	Endobronchial ultrasound-guided transbronchial needle aspiration
ENB	Electromagnetic navigation bronchoscopy
IP	Interventional pulmonology
MT	Medical thoracoscopy
NBI	Narrow-band imaging
Nd: YAG	Neodymium-doped yttrium aluminum garnet
PBAL	Protected bronchoalveolar lavage
PSB	Protected specimen brush
RAB	Robotic-assisted bronchoscopy
TBNA	Transbronchial needle aspiration
TLD	Targeted lung denervation
TTO2T	Transtracheal oxygen therapy
VAP	Ventilator-associated pneumonia
WLB	White light bronchoscopy
