# Correction: Reinoso et al. The Rise and Fall of Interventional Pulmonology Procedures: Lessons in Innovation, Evidence, and Abandonment. *Diagnostics* 2026, *16*, 1981

**DOI:** 10.3390/diagnostics16213537

**Published:** 2026-10-28

**Authors:** Jean Reinoso, Prince Ntiamoah, Avantika Nathani, Atul C. Mehta

**Affiliations:** 1 Division of Pulmonary, Allergy, Critical Care and Sleep Medicine, Emory University, Atlanta, GA 30322, USA; jreinos@emory.edu (J.R.); avantika.nathani@emory.edu (A.N.); 2 Division of Interventional Pulmonology, Aurora BayCare Medical Center, Green Bay, WI 54311, USA; prince.ntiamoah@aah.org; 3 Integrated Hospital Care Institute, Department of Pulmonary Medicine, Cleveland Clinic Foundation, Cleveland, OH 44195, USA

In the original publication [1], Figures 1, 2, 4 and 7–9 were published images with obtained copyright permission. Therefore, the captions of Figures 1, 2, 4 and 7–9 require reference citations, which should then be listed as references. 

Neff, T.A. Percutaneous Trephine Biopsy of the Lung. *Chest* **1972**, *61*, 18–23. https://doi.org/10.1378/chest.61.1.18 was already cited in main text; only missing citation from Figure 1 caption. This citation has now been inserted below Figure 1 in Section 2.1.1., “Trephine lung biopsy”, and should read as follows:

**Figure 1.** Trephine lung biopsy. A pneumatic drill-driven cutting needle used percutaneously to obtain cylindrical cores of lung parenchyma [8]. Reproduced with permission from Neff, T.A.; *Chest*; published by Elsevier, 1972.

Shaw, J.A.; Louw, E.H.; Koegelenberg, C.F. A practical approach to the diagnosis and management of malignant pleural effusions in resource-constrained settings. *Breathe* **2023**, *19*, 230140. https://doi.org/10.1183/20734735.0140-2023 was not cited. This citation has now been inserted below Figure 2 in Section 2.1.2., “Closed Pleural Biopsy”, and should read as follows:

**Figure 2.** Abrams’s closed pleural biopsy needle: the top one in the open position with the notch indicator on the grip in line with the specimen open notch (arrows). Below is the needle in the closed position, as the inner cylinder has been rotated down into the outer cylinder [15].

Meduri, G.U.; Beals, D.H.; Maijub, A.G.; Baselski, V. Protected bronchoalveolar lavage. A new bronchoscopic technique to retrieve uncontaminated distal airway secretions. *Am. Rev. Respir. Dis.* **1991**, *143*, 855–864. https://doi.org/10.1164/ajrccm/143.4_pt_1.855 was already cited in main text; only missing citation from Figure 4 caption. This citation has now been inserted below Figure 4 in Section 2.1.3., “Protected Specimen Sampling”, and should read as follows:

**Figure 4.** Protected bronchoalveolar lavage (PBAL): dual-lumen, balloon-tipped catheter that occludes a segmental bronchus for distal lavage and aspiration [30]. Reproduced with permission from Meduri, G.U., et al. *Am. Rev. Respir. Dis.*; published by Oxford University Press, 1991.

Chhajed, P.N.; Shibuya, K.; Hoshino, H.; Chiyo, M.; Yasufuku, K.; Hiroshima, K.; Fujisawa, T. A comparison of video and autofluorescence bronchoscopy in patients at high risk of lung cancer. *Eur. Respir. J.* **2005**, *25*, 951–955. https://doi.org/10.1183/09031936.05.00012504. was not cited. This citation has now been inserted below Figure 7 in Section 2.1.5., “Autofluorescence Bronchoscopy”, and should read as follows:

**Figure 7.** Autofluorescence bronchoscopy: (**A**) white-light view of endobronchial lesion (**B**) under blue-light illumination, normal mucosa fluoresces green while dysplastic or malignant epithelium appears red-magenta [49].

Leong, S.; Ju, H.; Marshall, H.; Bowman, R.; Yang, I.; Ree, A.-M.; Saxon, C.; Fong, K.M. Electromagnetic navigation bronchoscopy: A descriptive analysis. *J. Thorac. Dis.* **2012**, *4*, 173–185. https://doi.org/10.3978/j.issn.2072-1439.2012.03.08 was not cited. This citation has now been inserted below Figure 8 in Section 2.1.6., “Electromagnetic Navigation Bronchoscopy”, and should read as follows:

**Figure 8.** Electromagnetic navigation bronchoscopy (superDimension^®^) with extended working channel and locatable guide tracked against a pre-acquired CT roadmap [57].

Wang, Z.-R.; Yu, H.; Yang, B.; Gao, J.; Chen, R.-J.; Xu, Y.; Yang, Z.; Chen, L.-A. Probe-based confocal laser endomicroscopy imaging features of malignant lung nodules: results from a prospective in vitro study. *J. Thorac. Dis.* **2025**, *17*, 9131–9142. https://doi.org/10.21037/jtd-2025-1805 was not cited. This citation has now been inserted below Figure 9 in Section 2.1.7., “Confocal Laser Endomicroscopy”, and should read as follows:

**Figure 9.** Confocal laser endomicroscopy (CLE): (**A**) hematoxylin and eosin stain of invasive lung adenocarcinoma; (**B**) CLE probe providing real-time near-histologic “optical biopsy” images with thickened alveolar structural destruction [66].

The order of references has also been updated because of the addition of four references [15,49,57,66].

The authors state that the scientific conclusions are unaffected. This correction was approved by the Academic Editor. The original publication has also been updated.
