# Supplementary material for: The Rise and Fall of Interventional Pulmonology Procedures: Lessons in Innovation, Evidence, and Abandonment
Source: Diagnostics (Basel). 2026 Jun 25;16(13):1981. doi: 10.3390/diagnostics16131981 (PMC13360320; doi:10.3390/diagnostics16131981)
Supplement: Supplementary file 1 [file diagnostics-16-01981-s001.zip › diagnostics-4317804-supplementary.pdf]

Table S1: Rise and Fall of Diagnostic Interventional Pulmonology Procedures

Procedure	Era	Strengths	Limitations	Reason for Decline
Trephine Lung Biopsy	1960s–1970s	High diagnostic yield	High pneumothorax/bleeding	Replaced by CT-guided biopsy/Bronchoscopic biopsy
Closed Pleural Biopsy	1950s–1990s	Minimally invasive	Low yield in malignancy	Replaced by thoracoscopy
PSB/PBAL	1980s–2000s	High specificity	Affected by antibiotics	No outcome benefit vs non-invasive
c-TBNA	1980s–2000s	Low cost	Operator dependent	Replaced by EBUS-TBNA
AFB	1990s–2010s	High sensitivity	Low specificity	Replaced by NBI
ENB	2000s–2020s	Minimally invasive access	Variable yield	Competition from robotic bronchoscopy
CLE	2010s–present	Real-time imaging	Limited validation	Adjunctive only

Table S2: Rise and Fall of Therapeutic Interventional Pulmonology Procedures

Procedure	Era	Strengths	Limitations	Reason for Decline
TTO2T	1980s–2000s	Efficient oxygen delivery	Maintenance burden	Simpler oxygen systems
Bronchial Thermoplasty	2000s–2010s	RCT evidence	Procedural burden	Biologic therapies
Nd:YAG Laser	1980s–2000s	Immediate debulking	Fire risk, complexity	Replaced by APC/electrocautery
Airway Bypass	2000s	Strong physiology	No durability	Loss of patency
Lung Denervation	2010s	Mechanistic rationale	Modest benefit	Better medical therapy
Sealant/Vapor/Coils	2000s–2010s	Volume reduction	Inflammation, variable results	Insufficient efficacy and safety profile

References: Derived from cited studies within manuscript (EASE, STEP-UP, REVOLENS, AIRFLOW, BT trials).

AFB — Autofluorescence bronchoscopy
APC — Argon plasma coagulation
BAL — Bronchoalveolar lavage
BLD — Bronchoscopic lung denervation
BT — Bronchial thermoplasty
CBP — Closed pleural biopsy
CLE — Confocal laser endomicroscopy
COPD — Chronic obstructive pulmonary disease
CT — Computed tomography
c-TBNA — Conventional transbronchial needle aspiration
EBUS-TBNA — Endobronchial ultrasound-guided transbronchial needle aspiration
ENB — Electromagnetic navigation bronchoscopy
Nd:YAG — Neodymium-doped yttrium aluminum garnet
PBAL — Protected bronchoalveolar lavage
PSB — Protected specimen brush
RAB — Robotic-assisted bronchoscopy
TTO2T — Transtracheal oxygen therapy
VAP — Ventilator-associated pneumonia
